# Macrophage-based therapeutic strategies in glioblastoma: advancements in drug delivery and immunotherapy

**DOI:** 10.3389/fonc.2026.1746344

**Published:** 2026-02-05

**Authors:** Dongyang Li, Xinyue Zhang, Yanwei Du

**Affiliations:** 1 College of Integrated Traditional Chinese and Western Medicine, Changchun University of Chinese Medicine, Changchun, Jilin, China; 2 School of Nursing, Jilin University, Changchun, Jilin, China

**Keywords:** CAR-M therapy, glioblastoma, immune microenvironment, immunosuppression, immunotherapy, tumor-associated macrophages

## Abstract

Glioblastoma (GBM) is a highly aggressive brain tumor, characterized by extensive infiltration, neovascularization, and resistance to conventional therapies. The unique tumor microenvironment (TME) of GBM is shaped by the blood-brain barrier (BBB), immune cells, and glioma-derived factors, complicating treatment efficacy. Macrophages, particularly tumor-associated macrophages (TAMs), play critical roles in GBM progression through immune evasion, angiogenesis, and therapeutic resistance. Advances in macrophage-based therapies, including engineered macrophages (CAR-M) and macrophage-mimetic nanoplatforms, offer promising strategies for targeted treatment. These approaches leverage macrophages’ natural ability to cross the BBB and selectively accumulate in tumors, enhancing therapeutic outcomes. This review highlights the roles of macrophages in the GBM TME, recent developments in macrophage-based drug delivery systems, and the potential of CAR-M therapies for improving GBM treatment efficacy.

## 1 Introduction

Glioblastoma (GBM) is an aggressive and fatal brain tumor marked by extensive infiltration, neovascularization, and resistance to conventional therapies (1). Unlike systemic cancers, GBM resides in a unique neurovascular microenvironment, shaped by the blood-brain barrier (BBB), which controls interactions between tumor cells, immune cells, and molecular signaling networks (2). The tumor microenvironment (TME) includes macrophages, T lymphocytes, NK cells, and dendritic cells, with glioma-derived factors promoting immune evasion, tolerance, and angiogenesis (3–5). The infiltrative nature of GBM limits surgical resection, and the BBB hinders drug delivery, leading to poor treatment outcomes (6, 7).

Advances in nanobiotechnology have led to drug delivery systems inspired by biological membranes, exosomes, and synthetic interfaces (8, 9). These biomimetic vehicles offer benefits like enhanced biocompatibility and targeted delivery, but challenges remain in payload optimization and production scaling. Macrophages are promising for therapeutic delivery due to their phagocytic abilities, long circulation, and preferential migration to inflammation sites (10, 11). Their natural ability to cross the BBB and accumulate in tumors makes them ideal for GBM targeting (12). While CAR T-cell therapies have shown success in hematological cancers, their use in solid tumors is limited by immune suppression and poor tissue penetration (13). This has prompted exploration of CAR-engineered macrophages (CAR-M), which can infiltrate tumors, offering new therapeutic potential for GBM. This review discusses the role of macrophages in the GBM TME, the latest macrophage-based delivery systems, and the therapeutic promise of CAR-M strategies.

## 2 Macrophage activation and polarization

### 2.1 Activation of microglia

The TME in GBM is characterized by dense infiltration of glioma-associated myeloid cells (GAMs), comprising both CNS-resident microglia and bone marrow–derived macrophages (14, 15). GAMs constitute nearly half of the intratumoral cellular milieu, with approximately 85% originating from peripheral monocytes and macrophages, and the remaining 15% derived from resident microglia (16). Microglia, originating from yolk sac progenitors, function as the brain’s resident innate immune sentinels, distinguished by high expression of TMEM119, P2RY12, and CX3CR1 (17, 18). Under homeostatic conditions, they regulate neuronal–glial interactions and mediate early immune surveillance. In glioblastoma, microglia primarily engage in antigen presentation and secrete proinflammatory cytokines such as IL-1β and TNF-α. In contrast, bone marrow–derived monocytes are recruited by glioma-derived chemoattractants and subsequently differentiate into TAMs with immunosuppressive properties (19–21). These TAMs potently inhibit cytotoxic T cell activity, facilitate neovascularization, and produce IL-10, VEGF, and TGF-β, thereby sustaining tumor progression (22, 23). Transcriptomic analyses reveal that TAMs are enriched in M2-like markers, including CD206, CD163, and ARG1, distinguishing them from microglial populations (24, 25). The accumulation of these cells within an immunologically regulated environment plays a pivotal role in gliomagenesis and tumor progression. Microglial activation is induced by glioma-secreted factors like CSF-1 and CX3CL1, which lead to the upregulation of pro-tumorigenic and immunomodulatory molecules such as TGF-β, IL-1β, and EGF, thereby promoting enhanced tumor cell motility and growth (26, 27). Furthermore, the breakdown of the BBB facilitates the entry of TAMs from the bloodstream. Their recruitment is mediated by various glioma-derived chemokines, including CCL2, CX3CL1, CSF-1, GM-CSF, and EGF (28, 29). Upon infiltration into the tumor microenvironment, TAMs release a variety of pro-invasive and pro-angiogenic molecules, including MMPs, VEGF, PDGF, fibroblast growth factor, and inflammatory chemokines such as CXCL8 (30–32). TAMs demonstrate significant functional flexibility, with the ability to shift between a classically activated phenotype (M1) and an alternatively activated state (M2) in response to various microenvironmental stimuli (30, 33, 34). M1 polarization is triggered by signals such as IFN-γ, LPS, GM-CSF, and TNF-α, resulting in increased phagocytic activity and the release of proinflammatory mediators, including ROS, IL-1β, IL-6, IL-12, IL-23, and iNOS (35). These M1-dominant TAMs not only enhance dendritic cell activation and induce M2 macrophage repolarization toward an inflammatory phenotype but also contribute to tumor cell elimination through cytotoxic processes (32). M2 polarization is induced by cytokines like IL-4, IL-13, TGF-β, and M-CSF, leading to the upregulation of markers such as Arg-1, CD206, and CD163. TAMs polarized toward the M2 phenotype primarily secrete immunosuppressive and pro-tumorigenic factors, including IL-10 and chemokines CCL17, CCL18, and CCL22 (36, 37).

### 2.2 Reprogramming and plasticity of M1/M2 macrophage

In GBM, M2-skewed TAMs contribute to tumor growth and invasiveness by releasing factors such as MMPs, EGF, and VEGF (31, 38). They also promote the recruitment of FOXP3^+^ regulatory T cells, which dampen cytotoxic T cell function, while simultaneously remodeling the extracellular matrix to hinder CD8^+^ T cell infiltration either enzymatically or by physical barrier formation, thus fostering an immune-sheltered environment that supports tumor progression (39–41). The transition between M1-like and M2-like states is regulated by intricate signaling networks, particularly involving pathways such as JAK/STAT, MAPK, PI3K/AKT, NOTCH, and NF-κB (42–45). STAT1 phosphorylation plays a key role in promoting M1-like responses, whereas STAT6 activation drives M2 differentiation (46, 47). Specifically, GM-CSF combined with SHP2 inhibition enhances the expression of canonical M2 markers such as CD163, CD206, and Arg1, thereby reinforcing the M2 phenotype (48, 49). The acidic microenvironment, primarily driven by lactate accumulation, not only facilitates immune evasion but also activates HIF-1α signaling, which in turn promotes the transcription of genes such as VEGF and Arg1, hallmark markers of the M2 phenotype (50, 51). This polarization is blocked in HIF-1α-deficient models, highlighting the critical role of the lactate–HIF-1α axis in the induction of M2 macrophage polarization (51–53). Beyond lactate-induced HIF-1α stabilization, hypoxia and metabolic cues act in concert to shape TAM function in glioblastoma (41). In hypoxic tumor niches, HIF-1α and HIF-2α orchestrate transcriptional programs that not only promote angiogenesis via VEGF induction but also drive immunosuppressive macrophage polarization through Arg1, CD206, and IL-10 expression (34, 54). Hypoxia also suppresses oxidative phosphorylation while enhancing glycolysis in TAMs, creating a feedback loop that reinforces the hypoxic microenvironment (55, 56). Furthermore, tumor-derived metabolites such as succinate, adenosine, and kynurenine modulate TAM activity via mTOR and AHR signaling, linking metabolic checkpoints to immunological fate decisions. These signals synergistically reprogram macrophages toward an M2-like state, which contributes to glioma progression, vascular remodeling, and therapy resistance. Therapeutic strategies aimed at disrupting these hypoxia-metabolism–TAM circuits are currently being explored to restore antitumor immunity and improve treatment outcomes in GBM.

The cytokine TGF-β, which is secreted by both tumor cells and M2-like TAMs, further strengthens the immunosuppressive phenotype through Smad3-dependent signaling pathways (57–59). In contrast, interferon-γ activates STAT1 to drive M1-associated immune responses, whereas microbial ligands, including lipopolysaccharides, activate NF-κB signaling, promoting proinflammatory macrophage activation (60). GM-CSF stimulates STAT5, fostering M1 polarization, while IL-4 and IL-13 preferentially activate STAT6 to promote M2 differentiation (61). The presence of M2-like TAMs in tumors correlates with a reduction in TNF signaling and is associated with poor clinical prognosis (62). Novel therapeutic strategies for glioma aim at depleting M2-like TAMs, disrupting their immunosuppressive function, or altering their polarization. Molecules such as Arg1 and PD-L2, which are involved in immune suppression, are regulated by signals from the tumor microenvironment, and inhibition of TNF signaling has been shown to elevate M2-like cell frequency (15, 63). Additionally, glioblastoma demonstrates increased activation of the Wnt/β-catenin signaling pathway, along with a significant overexpression of its downstream effector WISP1. Experimental knockdown of WISP1 selectively reduces M2-like TAM populations without affecting M1 subsets, highlighting WISP1 as a critical regulator in sustaining the tumor’s immunosuppressive environment (64). Studies have demonstrated the feasibility of using cytokines and immune agonists to induce M2-to-M1 repolarization of TAMs in glioblastoma and other solid tumors. For instance, intratumoral administration of IFN-γ has been shown to enhance M1-like polarization and augment anti-tumor immune responses by increasing iNOS expression and antigen presentation capacity in preclinical glioma models (65). Similarly, GM-CSF has been utilized to promote proinflammatory TAM phenotypes, with some early-phase clinical trials (NCT00331526) suggesting immunologic reshaping of the tumor microenvironment in patients with gliomas (66, 67). In addition, TLR agonists—notably TLR3 (poly I:C) and TLR9 (CpG oligonucleotides)—have been tested for their ability to activate NF-κB signaling and reprogram TAMs toward a tumoricidal M1-like state (65, 68). These agents, either alone or in combination with immune checkpoint inhibitors, have shown promise in enhancing TAM plasticity and improving therapeutic efficacy.

## 3 Influence of M1 and M2 TAMs on GBM development

**Figure 1:**
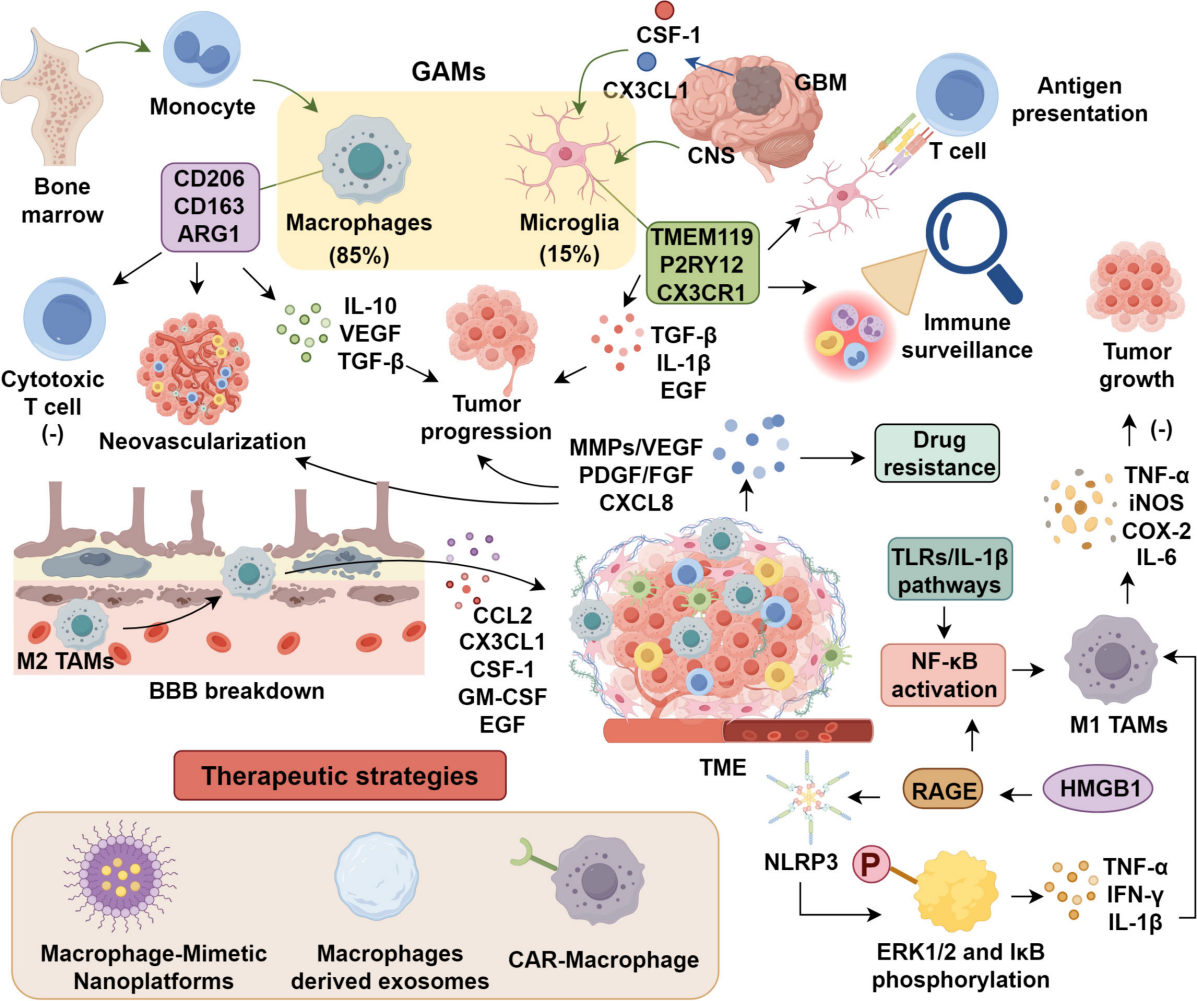
Roles of macrophages in glioblastoma progression.

TAMs are the primary immune cells infiltrating the GBM microenvironment, where they play pivotal roles in both tumor initiation and progression. These macrophages demonstrate considerable phenotypic flexibility, adopting either a pro-inflammatory M1-like or a more immunosuppressive M2-like phenotype (14). The M2 subset releases a range of bioactive factors, such as TGF-β, IL-10, VEGF, matrix metalloproteinases, and various chemokines, including CCL15, CCL17, and CCL22. Together, these factors promote angiogenesis, enhance stem cell-like traits in cancer cells, modify the extracellular matrix, facilitate immune evasion, increase resistance to treatments, and sustain the M2 phenotype (14, 33). Besides, M1-like TAMs are defined by their secretion of inflammatory cytokines, including TNF-α, IL-1β, IL-6, IL-8, IL-12, and IL-23. This profile supports Th1-type immune responses and amplifies the cytotoxic activities of NK cells (33). The activation of toll-like receptors (TLRs) or IL-1β signaling pathways triggers NF-κB activation, driving M1 polarization, which is characterized by an upregulation of TNF-α, IL-12, inducible iNOS, COX-2, and IL-6. While this M1 phenotype may inhibit tumor growth, it also induces significant inflammatory responses and contributes to increased cell death (69). Interestingly, NF-κB signaling is also implicated in facilitating metastatic progression driven by M2 macrophages (70, 71). Fibroblast-derived interleukin-33 promotes M2 polarization, and NF-κB–mediated MMP9 transcription enhances the invasive properties of cells (72, 73). The NF-κB–HIF-1 signaling axis is crucial for maintaining the M2 phenotype through the interaction between neoplastic cells and TAMs, which supports both angiogenesis and metabolic reprogramming (74, 75). HMGB1 initiates a cascade that is RAGE-dependent, involving NF-κB and NLRP3, with ERK1/2 and IκB phosphorylation driving the increased expression of proinflammatory cytokines, including TNF-α, IL-6, and CCL2, which enhances M1-like characteristics (76). Conversely, M2-polarized TAMs utilize the IL-10–JAK/STAT3 pathway, promoting tumor progression through immune suppression (77). Glioma-derived CSF-1 serves as a chemoattractant for microglia, directing them to an M2-like transcriptional program, whereas M1-polarized TAMs counteract this by neutralizing CSF-1R-mediated immunosuppression, thus boosting antitumor immunity and cytotoxic effects (78). Targeting M-CSF signaling pharmacologically has shown to significantly diminish the population of M2-type TAMs in the tumor microenvironment (79) (**Figure 1**).

## 4 Macrophage-driven strategies in glioblastoma therapy

### 4.1 Macrophage-mimetic nanoplatforms for targeted glioma delivery

Nanocarriers coated with cell membranes represent an innovative approach to drug delivery, blending synthetic interiors with natural membrane shells (80). This biohybrid design merges the engineered core’s controllability with the inherent biological properties of native cell membranes (81). The interior typically houses therapeutic agents, which are often engineered to release their contents upon exposure to specific tumor-associated stimuli (82, 83). Erythrocyte-derived membrane-coated nanoparticles had significantly extended retention times in circulation, thus paving the way for more advanced membrane-based drug delivery systems (84, 85). The progression of glioblastoma is characterized by extensive immune cell infiltration, particularly tumor-associated macrophages, which arise from both microglia in the brain and bone marrow-derived precursors (86). These macrophages exhibit significant plasticity, transitioning between pro-inflammatory (M1-like) and tumor-promoting (M2-like) phenotypes in response to signals from the tumor microenvironment (15, 20).

Macrophage-derived membranes offer a unique advantage for targeted drug delivery to gliomas. Specifically, integrin α4β1 mediates selective binding to vascular cell adhesion molecule-1 (VCAM-1), frequently upregulated on glioma cells, while α4 integrin and macrophage-1 antigen enhance translocation across the BBB (87, 88). This dual-targeting capability promotes both tumor-specific accumulation and BBB penetration. A representative approach involves a macrophage membrane-coated nanosystem comprising poly(N-vinylcaprolactam)-based nanogels co-loaded with cisplatin and MnO_2_, enabling glioma-targeted delivery, real-time magnetic resonance imaging, and synergistic chemo–chemodynamic therapy (89). To traverse the blood–brain barrier, researchers developed biomimetic nanoparticles (MDINPs) via extrusion, cloaking them with macrophage membranes (84, 90). Built on a DSPE-PEG scaffold and loaded with the NIR-Ib dye IR-792, MDINPs exhibited efficient brain penetration, glioblastoma-specific accumulation, and enabled high-resolution NIR-Ib imaging. In murine models, these particles achieved potent photothermal ablation of tumors, resulting in significantly prolonged survival (91). Composite coatings derived from macrophage membranes in combination with other cellular sources, such as platelets, cancer cells, red blood cells, or M1-polarized macrophages, have shown promising therapeutic efficacy. For example, a dual-membrane nanocarrier composed of neutrophil and macrophage membranes (NMm PLGA/RAPA) was constructed through sequential sonication and extrusion steps (92). This biomimetic system enabled passive traversal of the blood–brain barrier and facilitated microenvironment-responsive tumor targeting, yielding potent antitumor activity in preclinical glioblastoma models (**Table 1**).

**Table 1:** Therapeutic strategies targeting macrophages in glioblastoma (GBM).

Therapeutic strategy	Mechanism of action	Targeted macrophage phenotype	Targets	Challenges
Macrophage-Mimetic Nanoplatforms	Biohybrid nanoparticles utilizing macrophage membranes to enhance BBB penetration	M1 and M2 macrophages	Integrins (α4β1, α4), VCAM-1, Macrophage membrane proteins	Difficulty in optimizing payload release, scaling production, and achieving precise tumor targeting
CAR-Macrophage Therapy	Chimeric Antigen Receptor (CAR)-modified macrophages engineered for tumor targeting	M1-like (cytotoxic)	CD47, PD-L2, CSF1R, M-CSF	Risk of systemic adverse effects, limited understanding of antigen specificity and tumor infiltration
Macrophage-Targeted Exosome Delivery	Exosome-based drug delivery, utilizing macrophage-derived exosomes for cargo transfer	M1-like (immunogenic)	Doxorubicin, AS1411 aptamer, TGF-β, VEGF	BBB penetration, achieving sustained drug release, and targeting intracranial tumors effectively
Macrophage Polarization Modulation	Reprogramming M2-polarized TAMs into M1-like (tumoricidal) phenotype	M2 to M1 transition	IL-12, TNF-α, IFN-γ, GM-CSF, NF-κB	Potential off-target effects, efficient induction of M1 phenotype *in vivo*
Immune Checkpoint Inhibition	Blockade of immune checkpoints (PD-1, CTLA-4) to enhance macrophage-mediated immunity	M1-like (tumoricidal)	PD-1, PD-L1, CTLA-4, TGF-β	Overcoming resistance, limited efficacy in solid tumors, immune-related adverse events
Targeting Tumor-Associated Cytokines	Inhibition of pro-tumor cytokines (TGF-β, IL-10) to disrupt TAM function	M2-like (immunosuppressive)	TGF-β, IL-10, Arg-1	Cytokine specificity, potential for systemic toxicity
Macrophage-Targeted Nanogel Systems	Nanogel-based delivery systems engineered for macrophage targeting and tumor penetration	M1 and M2 polarizations	Cisplatin, MnO_2_, α4β1 integrin	Drug release optimization, biocompatibility, scalability of nanogel systems
Hypoxia-Driven Macrophage Targeting	Targeting macrophage polarization via hypoxic signaling pathways (HIF-1α)	M2-like (immune suppressive)	HIF-1α, VEGF, Arg-1, STAT3	Controlling hypoxia-induced polarization, off-target effects due to hypoxic conditions

### 4.2 Applications of exosomes originating from macrophages in glioblastoma therapy

Exosomes, defined as nanoscale extracellular vesicles enclosed by a lipid bilayer and with diameters of 30–150 nm, facilitate communication between cells (93). Their intrinsic ability to cross the blood–brain barrier, coupled with prolonged circulatory stability and low off-target distribution, underscores their potential as targeted delivery vehicles for central nervous system therapeutics (94). The functional properties and therapeutic relevance of exosomes are closely dictated by their cellular origin, as their molecular contents mirror the physiological state of the parent cells (95). Macrophage-derived exosomes have emerged as immunologically active nanovesicles enriched in MHC class II molecules, thereby enabling potent antigen presentation and modulation of immune responses (96). Their intrinsic tropism for tumor sites, coupled with an exceptional ability to traverse the blood-brain barrier, underscores the promise as precision delivery vehicles in cancer therapy (97). Notably, exosomes have been successfully engineered to encapsulate and transport chemotherapeutics such as doxorubicin, illustrating their translational potential as bioresponsive drug carriers (95). Although exosomes can traverse the blood–brain barrier, achieving sufficient drug accumulation within intracranial gliomas remains a formidable challenge, necessitating the development of exosome-based systems with enhanced targeting specificity (6, 94). Moreover, macrophage-derived exosomes were engineered with AS1411, a nucleolin-targeting aptamer, to facilitate glioblastoma-specific delivery (98, 99). These modified exosomes exhibited high affinity for glioma cells and achieved superior therapeutic efficacy relative to non-targeted nanocarriers. In an alternative approach, M1-polarized macrophage-derived exosomes were exploited to counteract glioblastoma progression (100, 101). Upon systemic administration and BBB penetration, these vesicles reprogrammed tumor-associated macrophages toward an M1-like phenotype, thereby reshaping the local immunosuppressive microenvironment. Within the tumor, hydrogen peroxide–induced CPPO chemiluminescence activated Ce6-mediated photodynamic generation of cytotoxic reactive oxygen species (102). Concurrent oxygen consumption facilitated the bioreductive conversion of AQ4N into its active form, AQ4. This combined strategy effectively integrates immune reprogramming, photo-initiated reactive species production, and hypoxia-dependent drug activation, producing a powerful anti-glioma response *in vivo* (103, 104).

### 4.3 CAR-macrophage

The impressive clinical outcomes achieved by chimeric antigen receptor (CAR)-engineered T cells against hematologic cancers have spurred interest in extending this immunotherapeutic strategy to solid tumors (105, 106). Translating CAR-T cell therapy into the solid tumor arena, however, faces considerable obstacles, such as poor tumor infiltration, an immunosuppressive tumor microenvironment, and potential off-tumor cytotoxicity, which collectively impair T cell activity (107, 108). A clinical study involving patients treated with CAR-NK cells did not observe serious adverse events (109). CAR-NK cells present certain benefits over their T cell counterparts, notably easier sourcing and a lower risk of neurological complications. Despite these advantages, issues related to the precision of antigen targeting, heterogeneity within NK cell populations, and scalable production persist (110). Given these constraints, macrophages are increasingly recognized as a promising platform for CAR engineering. Their intrinsic capacity for phagocytosis, antigen presentation, and activation of adaptive immunity allows them to function dually as cytotoxic agents and immune modulators, highlighting their potential to improve tumor penetration and treatment outcomes in solid malignancies (111, 112). Recent investigations into CAR-Ms have largely utilized cellular origins like THP-1 monocytic cell lines, peripheral blood mononuclear cells (PBMCs), or induced pluripotent stem cells (iPSCs). A notably advantageous source involves CAR-Ms differentiated from iPSCs (CAR-iMACs), which present significant potential for scalable production and are highly receptive to genetic modification (113–115). The development of CAR-iMACs specifically designed for anticancer immunotherapy. Initially characterized by an M2-polarized state, these engineered cells underwent a shift towards an M1-like phenotype following contact with tumor cells, consequently gaining strong phagocytic function and maintaining anti-tumor responses in living organisms (116, 117). A critical consideration is that translation to clinical practice is still nascent, as the majority of current research is limited to preliminary-stage clinical studies.

## 5 Conclusion

Macrophages, particularly TAMs, are pivotal in GBM progression, mediating various aspects such as immune evasion, angiogenesis, and resistance to conventional treatments. The plasticity of TAMs, with their ability to transition between M1 and M2 phenotypes, further complicates therapeutic strategies. M2-polarized TAMs, in particular, create a tumor-supportive microenvironment by secreting immunosuppressive cytokines and facilitating angiogenesis, which promotes tumor growth and metastasis. Targeting TAM polarization or reprogramming these cells into tumoricidal M1-like phenotypes holds significant therapeutic promise.

Advancements in nanobiotechnology have enabled the development of innovative macrophage-based drug delivery systems, such as macrophage-mimetic nanoplatforms and CAR-M therapies. These systems utilize the natural homing abilities of macrophages to target GBM tumors and cross the BBB, addressing key challenges in GBM treatment. The therapeutic potential of CAR-M cells, which harness the macrophage’s ability to phagocytose and present antigens, offers a novel approach for immune modulation in GBM therapy. While early-stage clinical trials show promise, the translation of these therapies to clinical practice requires further refinement to enhance specificity, scalability, and minimize potential off-target effects. Continued exploration of these strategies could significantly improve outcomes for GBM patients, providing a new avenue for combating this fatal disease.
